# CAFrgDB: a database for cancer-associated fibroblasts related genes and their functions in cancer

**DOI:** 10.1038/s41417-023-00603-4

**Published:** 2023-03-15

**Authors:** Qiang Yuan, Yi Chu, Xiaoyu Li, Yunshu Shi, Yingying Chen, Jimin Zhao, Jing Lu, Kangdong Liu, Yaping Guo

**Affiliations:** 1 Department of Pathophysiology, State Key Laboratory of Esophageal Cancer Prevention and Treatment, School of Basic Medical Sciences, Zhengzhou University, Zhengzhou, 450001 China; 2 China-US (Henan) Hormel Cancer Institute, Zhengzhou, Henan 450001 China

**Keywords:** Cancer microenvironment, Tumour heterogeneity

## Abstract

As one of the most essential components of the tumor microenvironment (TME), cancer-associated fibroblasts (CAFs) interact extensively with cancer cells and other stromal cells to remodel TME and participate in the pathogenesis of cancer, which earmarked themselves as new promising targets for cancer therapy. Numerous studies have highlighted the heterogeneity and versatility of CAFs in most cancer types. Thus, the identification and appropriate use of CAF-related genes (CAFGenes) in the context of specific cancer types will provide critical insights into disease mechanisms and CAF-related therapeutic targets. In this study, we collected and curated 5421 CAFGenes identified from small- or large-scale experiments, encompassing 4982 responsors that directly or indirectly participate in cancer malignant behaviors managed by CAFs, 1069 secretions that are secreted by CAFs and 281 regulators that contribute in modulating CAFs in human and mouse, which covered 24 cancer types. For these human CAFGenes, we performed gene expression and prognostic marker-based analyses across 24 cancer types using TCGA data. Furthermore, we provided annotations for CAF-associated proteins by integrating the knowledge of protein-protein interaction(s), drug-target relations and basic annotations, from 9 public databases. CAFrgDB (CAF related Gene DataBase) is free for academic research at http://caf.zbiolab.cn and we anticipate CAFrgDB can be a useful resource for further study of CAFs.

## Introduction

Mounting evidence shows that traditional treatment paradigms which predominantly target the tumor cells are usually not sufficient to root out their malignancy, as the tumor stroma or tumor microenvironment (TME) may hamper the efficacy of therapies and thus lead to therapeutic failure in clinical practice [1]. TME, as a complex system between tumor and their supporting stromal, including macrophages, endothelial cells, immune cell, vessels, extracellular matrix and fibroblasts, is depicted as the soil to participate in tumor initiation and progression and has drawn extensive attention in cancer research and therapy [2]. As a central component of the TME, cancer-associated fibroblasts (CAFs) can not only interact productively with cancer cells but also make a profound impact on the other components of the TME. Thus, CAFs have cemented themselves as a prominent functional role in modulating the efficacy of therapies and has evolved into a novel kind of therapy target.

**Fig. 1:**
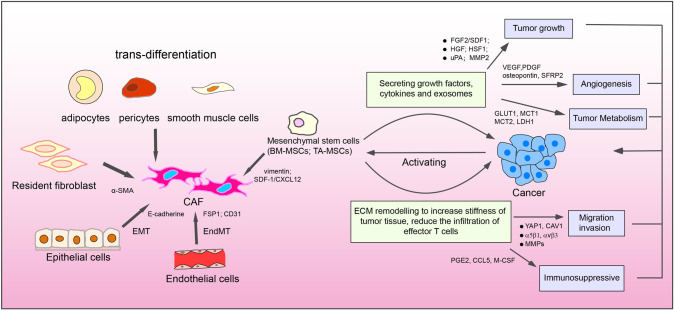
Origin of CAFs and summary CAF-mediated regulation of cancer biological behavior. Potential cellular sources of CAF including resident fibroblast, bone marrow-derived mesenchymal stem cells and tumor-associated MSCs, epithelial cells, endothelial cells, and trans-differentiation of cells (pericytes, adipocytes, smooth muscle cell). CAFs contribute to tumor progression through multiple mechanisms. CAFs induce EMT remolding, and produce a plethora of pro-tumorigenic factors to orchestrate the development of cancer through proliferation, angiogenesis, metastasis, changing metabolic state of the tumor and immunosuppression.

Emerging advances indicated that CAFs are complex populations with property of heterogeneity. On the one hand, the spectrum of origins contributes to explain the heterogeneity of CAFs. Resident fibroblast acquires a myofibroblast-like phenotype via transforming growth factor β (TGFβ) activation, accompanying with α-SMA expression [3]. Mesenchymal Stem Cells are one of the major sources of CAFs. Bone marrow-derived mesenchymal stem cells (BM-MSCs) can differentiate into CAFs, which feature a higher SDF1 expression [4]. In addition, tumor-associated MSCs (TA-MSCs) also act as potential CAF precursor cells with vimentin expression [5]. Furthermore, there is evidence that epithelial cells in the contest of cancer go through epithelial-to-mesenchymal transition (EMT) described as lower level of E-cadherin, and can contribute to become CAF [6]. Another source of CAF is endothelial cells, which can acquire CAF phenotypes via the endothelial-to-mesenchymal transition (EndMT), upregulating FAP1 and downregulating CD31 [7]. Finally, a small portion from trans-differentiation of cells can also convert into CAFs, further share fibroblast-like properties, including pericytes, adipocytes, smooth muscle cells [8, 9] (Fig. 1).

Reactive fibroblast shares the particular features, such as enhanced capacity of protein synthesis, proliferation and contraction [10]. In terms of structure and function, CAFs make crucial contributions to the biological behavior of tumor cells via versatile mechanisms. For instance, CAFs serve as synthetic machines that release large quantities of factors to involve in tumor proliferation, angiogenesis, tumor metastasis. TGFβ, Fibroblast growth factor 2 (FGF2), matrix metalloproteases 2 (MMP2) and hepatocyte growth factor (HGF) derived from CAF endow tumor cells with stronger proliferative behavior [1, 11–13]. Many other factors from CAFs, such as VEGF, PDGF SFRP2, osteopontin, can drive tumor angiogenesis [1, 14]. CAFs share a metabolic symbiosis with tumor cells to favor malignancy progression. CAFs utilize metabolic reprogramming through “Warburg Effect” to promote malignant phenotype of cancer cells [15]. MCT1, MCT2, LDH1 and so on are found as higher expression in CAFs [16, 17]. Meanwhile, CAFs also remodel the extracellular matrix (ECM) structure to guide cancer cell to invade. CAFs secrete matrix MMPs to affect ECM stiffness, and YAP1 or CAV1 overexpression in CAFs can highly induce directional migration and invasiveness of carcinoma cells [12, 18, 19]. CAFs also build up ECM structure through αvβ3 integrin and α5β1 integrin to support cancer cell [20]. On the other hand, ECM remodeling by CAFs forms a physical barrier against the immune system. CAFs produce some factors, such as CCL5, PGE2, M-CSF, to have an important role in immune evasion [1, 5, 21] (Fig. 1). Although increasing evidences suggested CAFs have negative regulation in cancer, the exact mechanism needs to illustrate.

Diversity of cellular sources manifests the heterogeneity of CAF. Such heterogeneity also is ascribed to the distribution of biological markers and cancer types, which is described as a strategy to at least partially contribute to distinct CAF subpopulations [22]. α-smooth muscle actin (α-SMA), fibroblast activation protein (FAP), fibroblast specific protein 1 (FSP1, also termed as S100A4) and platelet-derived growth factor receptor-α/β (PDGFRα/β) are considered as marker to define CAFs [23]. However, the identification of these candidate markers has been challenging due to the fact that other stromal cell populations also shared [24]. Hence, to understand their biological roles, CAFs subpopulation is divided into different subtypes according to the type of cancer and the distribution of markers [25]. Although CAFs subpopulation improved understanding of CAFs heterogeneity and functional diversity, targeting heterogeneity therapies has remained many challenges. Thus, there is an urgent need to understand functional heterogeneity of CAFs in depth.

In this study, we collected and curated 5421 CAFGenes. According to their function and distribution, we annotated them in detail. Owing to different roles in CAFs, these proteins are divided into three types, respectively named regulator, responsor and secretion. In addition, we provided rich annotations for CAFs-related proteins by integrating the knowledge from 9 additional resources that covered many aspects including the knowledge of cancer-associated information, protein-protein interaction(s), drug-target relations and basic annotations. The CAFrgDB database will be continuously updated and could also serve as a valuable tool to understand CAFs function.

## Methods

### Data collection and curation, classification

**Fig. 2:**
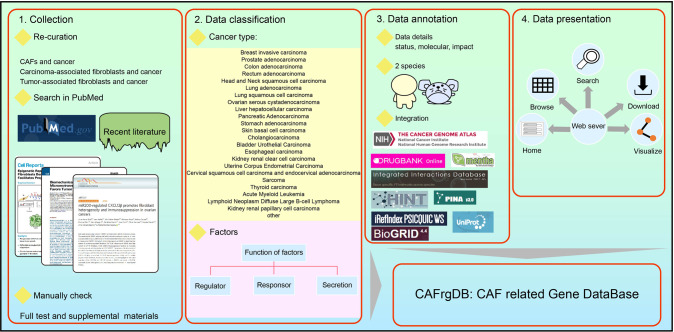
Overview of CAFrgDB. Data were collected from published literature in PubMed through manually check. We classified all collected proteins into 3 categories including responsor, regulator and secretion from 24 cancer type. A total of 5421 CAF-associated proteins are annotated in detail from human and mouse. Further, we further integrated annotations in 9 public data resources covering 5 aspects: protein function, interacting proteins, FDA-approved drugs, expression and prognosis in TCGA. Eventually, all entry were obtained with reliable evidence and CAFrgDB database was constructed. Users can access these different categorized annotations by clicking the picture.

In CAFrgDB 1.0, we manually collected 5421 CAFGenes together with their exact functions from the literature. To obtain known CAFGenes from the literature, we manually searched the PubMed database by a set of topically relevant keywords, such as “cancer-associated fibroblasts and cancer”, “CAFs and cancer” and “tumor-associated fibroblasts and cancer”. Totally, 13,621 entries are recorded involved in influencing the function of CAFs from 24 cancer type. For each literature, the information regarding samples was extracted. In addition, we directly retrieved other pertinent information such as cancer type, expression of CAF-related factors, experimental conditions along with the regulation mode of each factor from the research articles. Labels ‘increase’ or ‘decrease’ were used to indicate up-regulation or down-regulation of the regulation factors that enhance CAFs function (Fig. 2).

Notably, all collected genes were classified into secretion, regulator or responsor. Secretion was defined as the proteins which were secreted by CAFs. For example, FGF2, a well-characterized CAF-secreted protein contributes to tumor cell growth [26]. Thus, some factors or cytokines deriving from CAFs are categorized into “secretion”. In addition, it has been discovered that various tumor-derived stimuli signals can activate quiescent fibroblasts to become CAFs, which include soluble factors produced by the tumor and immune infiltrate such as TGFβ family ligands, lysophosphatidic acid (LPA), FGF, platelet-derived PDGF and interleukin-1 (IL-1), and here we classified the above of these proteins as regulator. Furthermore, a large number proteins were identified to be participated in CAF-mediated cancer progression. CAFs act as highly heterogeneous stromal cells, the interaction between CAFs and cancer cell supports tumorigenesis in various ways, especially signaling pathways. Meanwhile the crosstalk also medicates the biological behaviors of CAFs [27]. CAFs promote cancer progression through activation signal cascades including PI3K/AKT/mTOR signaling pathway, TGF-β signaling pathway, MAPK signaling pathway, etc [28]. Several major signal cascades involving in CAFs tumor-promoting or tumor-restraining phenotype, named as responsor. Responsor is recognized as a range of genes executing CAFs phenotype. These three components are not independent of others, and there is some overlap between them. Of course, these molecules might play entirely different roles in CAF from different cancer, which also reveals heterogeneity of CAFs.

### Experimental conditions

Experimental conditions include cancer types or cell lines, eukaryotic species, which are extracted from the literature. In CAFrgDB database, diverse cell lines from the same tissue also are recognized as parallel samples. To explore the relationship between CAFs and cancer, we also integrated transcriptome, proteomics and secretome from literature into our dataset. Meanwhile, we recorded the expression signature corresponding to their exact effect such as promotion or inhibition in CAFs.

### Protein basic and annotation information

After filtering for duplicates and reclassifying cancer subtypes, CAFrgDB encompassed 13,621 entries involving 24 cancer subtypes. For each study, we recorded how the CAFGenes regulate or effect the function of CAFs or act as secretor derived from CAFs in different cancer type. To enrich the data and facilitate users, we also integrated UniProt [29] and The Cancer Genome Atlas (TCGA) database [30] to annotate the CAFGenes. The basic information contains gene name, protein name, organism, UniProt ID, protein sequence, and function. According to the role in CAFs, we described these items as different CAFs type. More importantly, we provided detailed evidence for these factors in CAF including cancer type and regulation mode. Further, we used UniProt database to present annotation of the sequence and function of factors [31]. TCGA is currently the largest available dataset for deep sequencing for cancer patients [32]. The CAFrgDB exhibits various genome-wide data including gene expression data and clinical data from TCGA database, which provides clinical value for cancer research. We also depict the interaction partners in CAFs by compiling and integrating protein-protein Interaction database including IID [33], iRefIndex [34], PINA [35], HINT [36], Mentha [37] and BIOGRID [38]. Because targeting CAF heterogeneity has been expected to be a novel therapy for cancer, we integrated the information of Drugbank [39] (Fig. 2).

### Enrichment analysis based on CAFGenes

Two-sided hypergeometric test was adopted for enrichment analysis of each type of CAFGene based on the annotations of KEGG. For each type, we defined the following:

*N* = number of human proteins annotated by at least one term

*n* = number of human proteins annotated by term t

*M* = number of CAFGenes in each type annotated by at least one term

*m* = number of CAFGenes in each type annotated by term t

Then, the enrichment ratio was computed, and the *p*-value was calculated based on the hypergeometric distribution as below:

$$Enrichment\;ratio = \frac{{\frac{m}{M}}}{{\frac{n}{N}}}$$

$$p - value = \mathop {\sum }\limits_{m^\prime = m}^n \frac{{\left( {\begin{array}{*{20}{c}} M \\ {m^\prime } \end{array}} \right)\left( {\begin{array}{*{20}{c}} {N - M} \\ {n - m^\prime } \end{array}} \right)}}{{\left( {\begin{array}{*{20}{c}} N \\ n \end{array}} \right)}}\left( {Erichment\;ratio \ge 1} \right)$$

or

$$p - value = \mathop {\sum }\limits_{m^\prime = 0}^m \frac{{\left( {\begin{array}{*{20}{c}} M \\ {m^\prime } \end{array}} \right)\left( {\begin{array}{*{20}{c}} {N - M} \\ {n - m^\prime } \end{array}} \right)}}{{\left( {\begin{array}{*{20}{c}} N \\ n \end{array}} \right)}}\left( {Erichment\;ratio\, < \,1} \right)$$

### Web interface implementation

All the analysis results and entry information were curated using MySQL tables (https://www.mysql.com/cn/). The web interfaces of CAFrgDB were implemented in PHP + MySQL + JavaScript. Furthermore, we inlayed multiple retrieval mode and allowed the users to search for and download the corresponding information. All datasets and annotations can be downloaded at: http://caf.zbiolab.cn/download.php.

## Results

### Overview of CAFrgDB

**Fig. 3:**
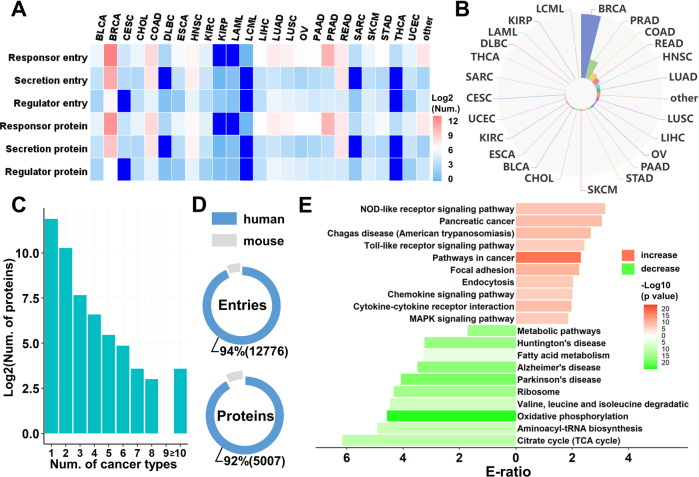
The statistics and analysis of the dataset. **A** Numbers of responsor, regulator and secretor in different cancer. **B** Numbers of CAF-related factors in different cancer type are summarized. **C** The distribution of numbers of CAF-entry in different cancer. **D** Comparison the number of entries from human and mouse. **E** The KEGG-based enrichment analysis of the CAF-related proteins.

CAFrgDB serves as the first literature-based database of the relations between CAFs and cancer. To establish CAFrgDB, we first collected all reported CAFGenes and their functions from the literature. After manual check of the full text and supplementary materials, 13621 entries involving 24 cancer types were obtained. Detailed information including cancer type or cell lines used in experiments, factors, the status of factors in CAFs and evidence of the relations were provided in the Webserver. In the current version of database, these entries were divided into 3 groups following the classification scheme from CAFrgDB. More specifically, we divide 13621 entries into three categories including 4982 responsors that participate in the function of CAFs to cancers, 1069 secretions that are secreted by CAFs and 281 regulators that contribute in modulating CAFs in human and mouse (Fig. 2). As shown in Fig. 3A, the distribution of three CAF type in different cancer is described. For example, totally 9352 entries have been implicated in breast cancer, all of which 114 are secretions, 1723 entries are regulators, and up to 7515 proteins acting as responsor that execute the function of CAFs. Presently, the largest number of CAF entries observed is for breast cancer, which encompasses 69% of all entries in our dataset, followed by prostate cancer (9%), colon cancer (4%) (Fig. 3B) (Table S1). In Fig. 3C, we summarized each of CAF-related entries involved in diverse cancer progression. The results indicated that only a small portion of protein factors are prone to involve in multiple cancers. However, notably, these proteins are more specific, and we found that more than 50% of known entries were only detected in one cancer type, suggesting they might be candidate CAF proteins for explanation the heterogeneity of CAFs. Only 11 proteins participate more than 10 types of cancer, including IL6, MET, IL8, CCL2, VEGFA, AKT1, AKT2, STAT3, SDF1, ACTA, SEPR (Table S2). In our dataset, known CAFGenes were collected from 2 eukaryotic organisms, including Homo sapiens and Mus musculus. In total, there were 12776 (92%) entries of 5007 (94%) proteins in human, and the result indicated that the most of CAFGenes-related experiments were conducted in human (Fig. 3D).

### Functions of CAFGenes

The status of factors in CAFs includes increase and decrease, and an enrichment analysis was performed based on the information. The top 10 mostly enriched KEGG pathways were chosen and visualized. These increasing-factors were enriched in NOD-like receptor signaling pathway (hsa04621), Toll like receptor signaling pathway (hsa04620), and Pathways in cancer (hsa05200). Furthermore, decreasing-factors were enriched in metabolic pathways (Fig. 3E).

Using the 5007 human CAFGenes belonged to 3 categories, we performed an enrichment analysis for each category of CAFGenes based on Kyoto Encyclopedia of Genes and Genomes (KEGG) annotations with the hypergeometric test [40, 41]. The mostly enriched KEGG pathways were chosen and visualized for each of these three categories. For the responsors, we observed that they were significantly enriched in cancer metabolism-associated processes, such as Spliceosome (hsa03040), Ribosome (hsa03010) and Oxidative phosphorylation (hsa00190). In addition, cancer-associated pathways including Pathways in cancer (hsa05200) and Citrate cycle (TCA cycle) (hsa00020) were also significantly enriched in responsors. The results were highly consistent with experimental studies [42, 43]. For the secretions, such as Focal adhesion (hsa04510), ECM-receptor interaction (hsa04512), Regulation of actin cytoskeleton (hsa04810), Antigen processing and presentation (hsa04612) and Complement and coagulation cascades (hsa04610) were enriched and demonstrated that these CAFGenes were highly involved in ECM remolding, which underlines the impact that CAFs present the ability to alter the surrounding environment via allowing the tumor matrix to be remolded [44]. Further analysis of regulators indicated that they were highly involved in regulating various types of signal transduction associated processes including TGF-beta signaling pathway (hsa04350), Cytokine-cytokine receptor interaction (hsa04060) together with NF-kappa B signaling pathway (hsa04064) and immune-associated processes such as Toll-like receptor signaling pathway (hsa04620), NOD-like receptor signaling pathway (hsa04621) and Chemokine signaling pathway (hsa04062) (Table S3). Notably, these results were not only highly consistent with previous studies [45, 46], but also provided valuable information for further deciphering versatile roles of human CAFGenes. It further provided clear evidence that CAFGenes play key roles in the response to cancer development, which indicated that CAFs have great potential to become target for cancer therapy.

### A comprehensive annotation of CAFGenes

We constructed CAFrgDB as a gene-centered database, and a variety of basic annotations, such as protein/gene names/aliases, UniProt accession numbers, functional descriptions and protein sequences were obtained from UniProt databases. For each known CAFrgDB-related protein, brief descriptions on its roles in CAF were present, and we termed “CAF type” including regulator, responsor and secretion. In addition, CAFrgDB presented two aspect evidences derived from low-through experiment and high-through experiment. In particularly, for each known CAFGenes, descriptions on available assays of sets of experiments were also summarized. Corresponding cancer type or cell lines for experimental analyses were supported, as well as users can acquire raw resource via PMIDs of primary references.

Moreover, importantly, by integrating the knowledge of 9 additional databases, we further annotated CAFGenes from 5 aspects. CAFrgDB database also provided specific annotations, for instance mRNA expression profiles in TCGA and prognosis of patients was also evaluated from TCGA in different cancer. We presented the analysis of expression level of 4974 CAFGenes transcripts from TCGA database and found total more than 30% genes (|log2(Foldchange)| > 1 and *p*-value < 0.05) showed significantly differential expression between cancer samples and adjacent normal samples at least in one cancer type. Furthermore, we also provided clinical association analysis about relationship between gene expression and patients survival, which accounts for more than 90% (Logrank test, *p* < 0.05) CAFGenes expression could indicated the prognosis of the patients at least in one cancer type. Herein, we developed comprehensive database, which included information on numerous PPIs in the BIOGRID, PINA, HINT, IID, MENTHA and iRefIndex database [46], corresponding to CAFGenes interactions, thus providing extensive information regarding CAF function. In addition, more than 90% CAFGenes have specific regulator network. In order to improve clinical utility, we also integrated the information of DrugBank and 1280 CAFGenes have FDA-approved drugs for further research reference.

### Web design and interface

**Fig. 4:**
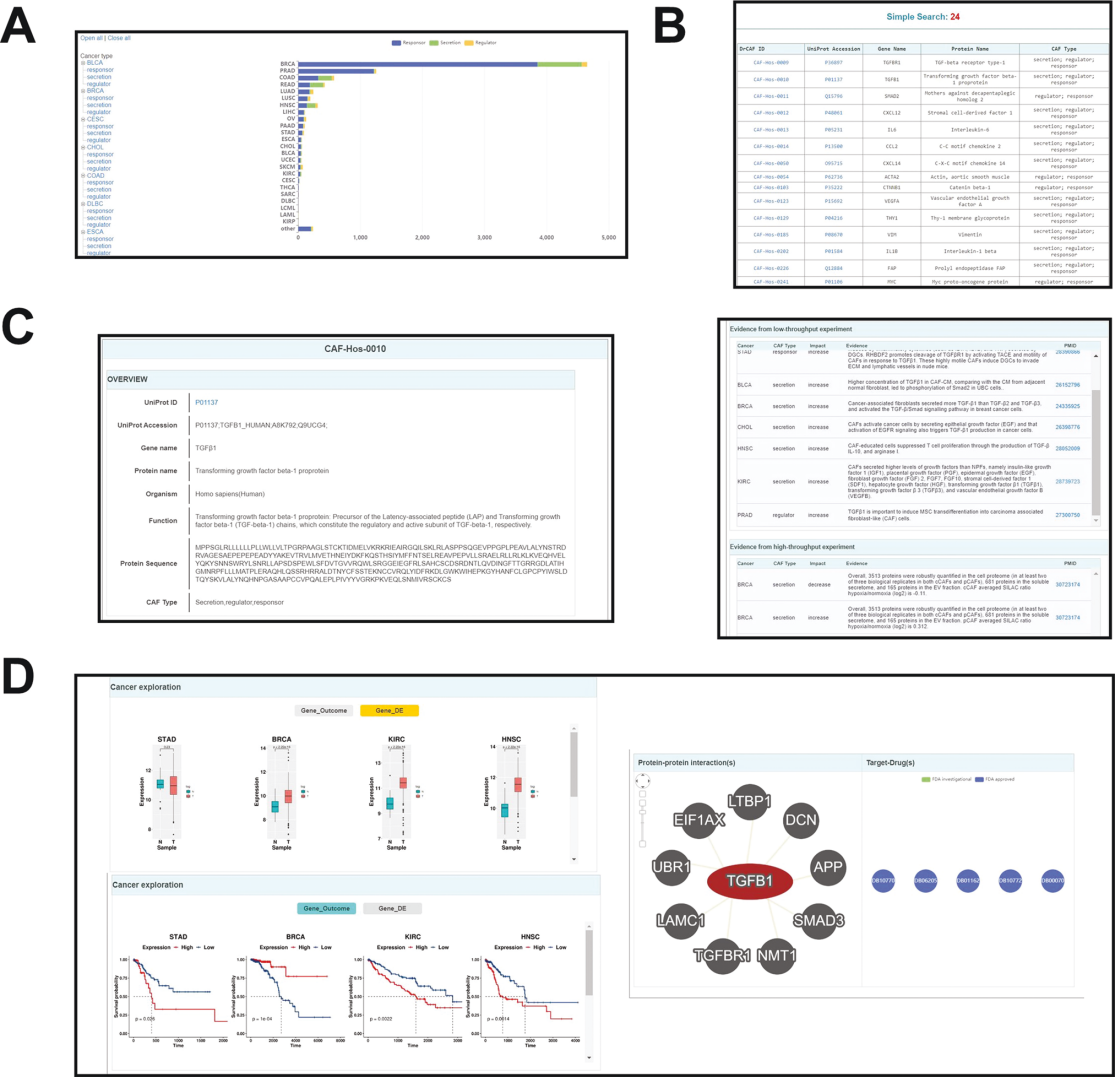
Overview of the CAFrgDB web interface. **A** The interface for data browsing. **B** Browse page for CAF type. **C** Basic annotation page of human TGFβ1 in CAFrgDB databse. **D** Detailed information regarding TGFβ1 in the other database.

CAFrgDB website feature a freely available and user-friendly interface for users to explore CAFs data within different cancer. The website comprises four sections: ‘Home’, ‘Browse’, ‘Search’, and ‘Download’. On the ‘Home’ page, users can acquire an overview of CAFs, including the total number of CAFs-related factors in different cancer from human and mouse. Based on the function, we classified factors into three categories including responsor, regulator and secretor, termed as CAF-related genes. For browsing the data in CAFrgDB, we implemented two options, including ‘Browse by CAF types’, and ‘Browse by cancer type’ (Fig. 4A). By clicking a specific cancer type, information of CAF-related proteins is shown in a table (Fig. 4B). Each entry in CAFrgDB database has a unique identification, named CAFrgDB ID, and we provided gene name, protein name, description from Uniprot and CAF type. Here, we selected the human TGFβ1 protein as an example to depict the usage of CAFrgDB. For browsing the data in CAFrgDB, we implemented two options, including ‘Browse by cancer types’ and ‘Browse by CAF type’. In the option of ‘Browse by cancer types’, users can click the cancer type of ‘ESCA’ under the cancer types super-class to browse all known CAFGenes involved in ESCA (Fig. 4A). Since human TGFβ1 is a known regulator for CAFs, users can also directly click ‘regulator’ in the option of ‘Browse by CAF types’, and then the result will be displayed in a table of the returned page for users to view all human CAF regulators (Fig. 4B). By clicking “BLCA”, “regulator” and ‘CAF-Hos-0010’, the final page of human TGFβ1 will be shown (Fig. 4C). In the gene page, basic annotations such as protein/gene names, Uniprot gene/protein IDs, and CAF type can be viewed (Fig. 4C left). The two aspect evidences derived from low-through experiment and high-through experiment of CAFGenes and its primary references PMIDs were also be linked (Fig. 4C right).

As for additional fundamental annotations, we compiled and integrated the knowledge from 9 additionally public resources. For additional annotations, user will obtain three modules including “cancer exploration”, “protein-protein interaction” and “target-drug”. In “cancer exploration” section, users can click the “GeneDE” botton to obtain multiple barcharts displaying the gene expression levels among different tumor tissues (Fig. 4D left). Besides, by clicking the ‘Gene Outcome’ tag, all survival analysis of this CAFGenes based on Kaplan–Meier method will also be displayed (Fig. 4D left). We also designed a “protein-protein interaction” module to construct synergistic regulatory network online by integrating three protein-protein interaction databases including IID, PINA and HINT. Furthermore, the database provided some FDA-approved drugs targeting the protein in “target-drug” section (Fig. 4D right). Specially, the annotation datasets can be freely downloaded at http://caf.zbiolab.cn/download.php. These interesting findings will benefit users that are devoted to investigating the regulatory mechanisms of CAFs. The three options for browsing the database, we also provided several options, including ‘Simple Search’, ‘Batch Search’ and ‘Advance Search’, for searching the data in CAFrgDB (http://caf.zbiolab.cn).

## Discussion

Malignant behavior of tumor cells is partially determined by TME and CAF is a cell type of paramount importance for TME with diverse functions, including secreting growth factors or cytokines and remodeling the tumor stroma to impact tumor malignancy [27, 47]. Thus, numerous evidence proposed a new cancer treatment paradigm, targeting CAFs [48, 49]. TME elicit a wide spectrum of dynamic alterations in cancer development, and CAFs have important roles in cancer pathogenesis within TME. Based on complex of TME and partially lack of specific CAF cell surface marker, precisely targeting CAFs has obtain some therapy effect along with side-effect [50–52]. Several major signal pathways affect not only the biological functions of CAFs but also the crosstalk between CAFs and cancer cells, even they also participate in malignant behaviors of cancers. Thus, it seems more feasible to target activation signaling and downstream effectors of CAFs. Owing to the complex inter-cellular interactions and regulator systems involving CAFs in the tumor stroma, it is hard to precisely target CAFs without influencing other cell populations and it is reasonable to be forced on cancer cells. Therefore, in this study, we summarized CAF-related gene and evaluated their expression profile in different cancer types via TCGA database, and tried to find these factors sharing tumorigenic phenotype and function in CAFs. We considered that targeting these factors not only might inhibit the cancer cell, more importantly convert TME into tumor-suppressive microenvironment. In order to speed up the development to clinic, we also integrated the DrugBank information, which provided rich annotations for therapy strategy.

Distinct sets of biomarkers and different classification strategies have been used to paraphrase diverse CAF subpopulations in different cancer types [53]. Particularly, emerging evidence provided a detailed characterization of CAFs in PDAC. There is lower expression of α-SMA and higher expression of inflammatory markers IL-6 and LIF, which are termed inflammatory CAFs (iCAFs) [3, 54]. Although iCAFs are more distal to cancer cells, they share immunomodulating secretome to involve in tumorigenesis. Another CAF populations are myofibroblasts (myCAFs), which is defined higher-SMA levels and exhibits myofibroblastic phenotypes. Compared with iCAFs, myCAFs are adjacent to tumor foci [55, 56]. In addition, CAFs subpopulation is possibly caused by marker expression or gene profiling. In colorectal cancer, according to gene expression and marker profiling, CAFs is identified with two distinct subpopulations, CAF-A and CAF-B. ACTA2, TAGLN and PDGFA serve as markers for CAF-B, whereas CAF-A cells specially express MMP2, DCN and COL1A2 [22]. Functionally, CAF-A cells are confirmed to express rebuilding ECM-related proteins, while CAF-B cells express cytoskeleton-related genes [57]. The heterogeneity of CAFs also present heterogeneous functions on cancer or produce a range of tumor-promoting (pCAFs) or tumor-restraining (rCAFs) effects in diverse cancers [58]. Although CAFs subpopulation highlights the requirement for studying CAFs in physiologically function, CAFs exhibit molecular and functional heterogeneity in different cancers and even at different stages of the same cancer. Therefore, any therapeutic strategies for targeting CAFs should exploit the specificity and diversity of CAFs.

With the development of single-cell RNA sequencing (scRNA-seq), scRNA-seq analysis has provided a deeper understanding of CAF heterogeneity with diverse putative functions in different cancer types, which has been performed to define different potential subpopulations of CAFs. For example, in lung cancer, clusters 2 highly expressing ACTA2 involved in angiogenesis; cluster 1 are enriched in tumor with strongly ECM proteins; clusters 4 are enriched at the leading edge of tumor mass; clusters 5 and clusters 7 possess similar high mTOR gene signature, but they are different from location distribution [53, 59]. Similarly, four CAFs subpopulations are identified in breast cancer [60–62]. Furthermore, scRNA-seq has been conducted to reveal heterogeneous alterations including tumor-promoting phenotype from non-invasive intraductal papillary mucinous neoplasms (IPMNs) to PDAC. During the progression of PDAC, CAFs subpopulation can shift between myCAFs and iCAFs. During non-invasive dysplasia to invasive cancer, myCAFs proportion is increasing from LGD-IPMNs to HGD-IPMNs, but in invasive cancer iCAFs is in a dominant position [63].

Recently, mounting studies for targeting CAFs have been explored in preclinical as below. Firstly, Directing CAF depletion via cell surface markers. FAP and αSMA act as marker of CAFs, have also been used to pharmacologically deplete CAFs. In PDAC, selective depletion of the α-SMA + myofibroblasts suppressed cancer metastasis [64]. A number of preclinical studies have been reported that specific depletion of FAP-expressing CAFs a or pharmacological inhibition of FAP also has shown promising antitumor activities [65]. αFAP-PE38 as FAP-targeting immunotoxin presents attractive tumor suppression in breast cancer mouse model [50]. Secondly, reprograming CAFs to quiescent state. CAFs simultaneously possess tumor-promoting and tumor-restraining function, the transfer function depending on TME. Therefore, strategy would be designed to tumor-promoting CAFs population or inducing tumor-promoting CAFs into quiescent state or even tumor-restraining phenotype. One of the examples of such an approach is supported by providing all-trans retinoic acid (ATRA) in pancreatic cancer. ATRA reset the fibroblasts to an inactive state and leads to reduced tumor growth via inhibiting Wnt–Catenin Signaling [66]. Targeting ECM proteins. Several clinical trials have been evaluated chemotherapeutic agents targeting Hyaluronan (HA) derived from CAFs in PDAC and gastric cancer. Currently, pathway inhibitors including JAK inhibitors [67], PDGFR inhibitors [68] and so on have conducted in clinical trials. In this study, we summarized all proteins involving in CAFs function and evaluated their expression profile in different cancer types via TCGA database, which might help optimize combination therapy strategy for target CAFs.

In summary, CAFrgDB hosts 13,621 entries of CAFGenes, which covered 24 cancer types. Furthermore, we provided annotations for CAF-associated proteins by integrating the knowledge of basic information, cancer-associated information, protein-protein interaction(s), drug-target relations and basic annotations, from 9 public databases. The database serves as a useful resource to help understanding the heterogeneity of CAF and such an integrative annotation supports valuable information for researchers. In the future, we will attempt to continuously maintain and update CAFrgDB by collecting and annotating newly identified CAFGenes and their exact functions in cancers. It should be noted that new functions might be reported for existing proteins in CAFrgDB, and their CAF types and the classification information will also be refined. We anticipate CAFrgDB can serve as a useful resource for further study of CAFGenes.

## Supplementary information

CAF entries were observed in different cancer types.

The number of CAF-related proteins participated in different cancer types.

The mostly enriched KEGG pathways were chosen and visualized for responsor,secretion and regulator.

## Data Availability

All data generated and analyzed during this study can be download at http://caf.zbiolab.cn/download.php.
